# Supplementary material for: The interactome of intact mitochondria by cross-linking mass spectrometry provides evidence for coexisting respiratory supercomplexes
Source: Mol Cell Proteomics. 2017 Dec 8;17(2):216–32. doi: 10.1074/mcp.RA117.000470 (PMC5795388; doi:10.1074/mcp.RA117.000470)
Supplement: Supplemental Data [file supp_RA117.000470_133922_0_supp_23978_fzffwf.zip › spectra_annotation/mito_DR_spectra_annotation/171-1-2-1-16-1.pdf]

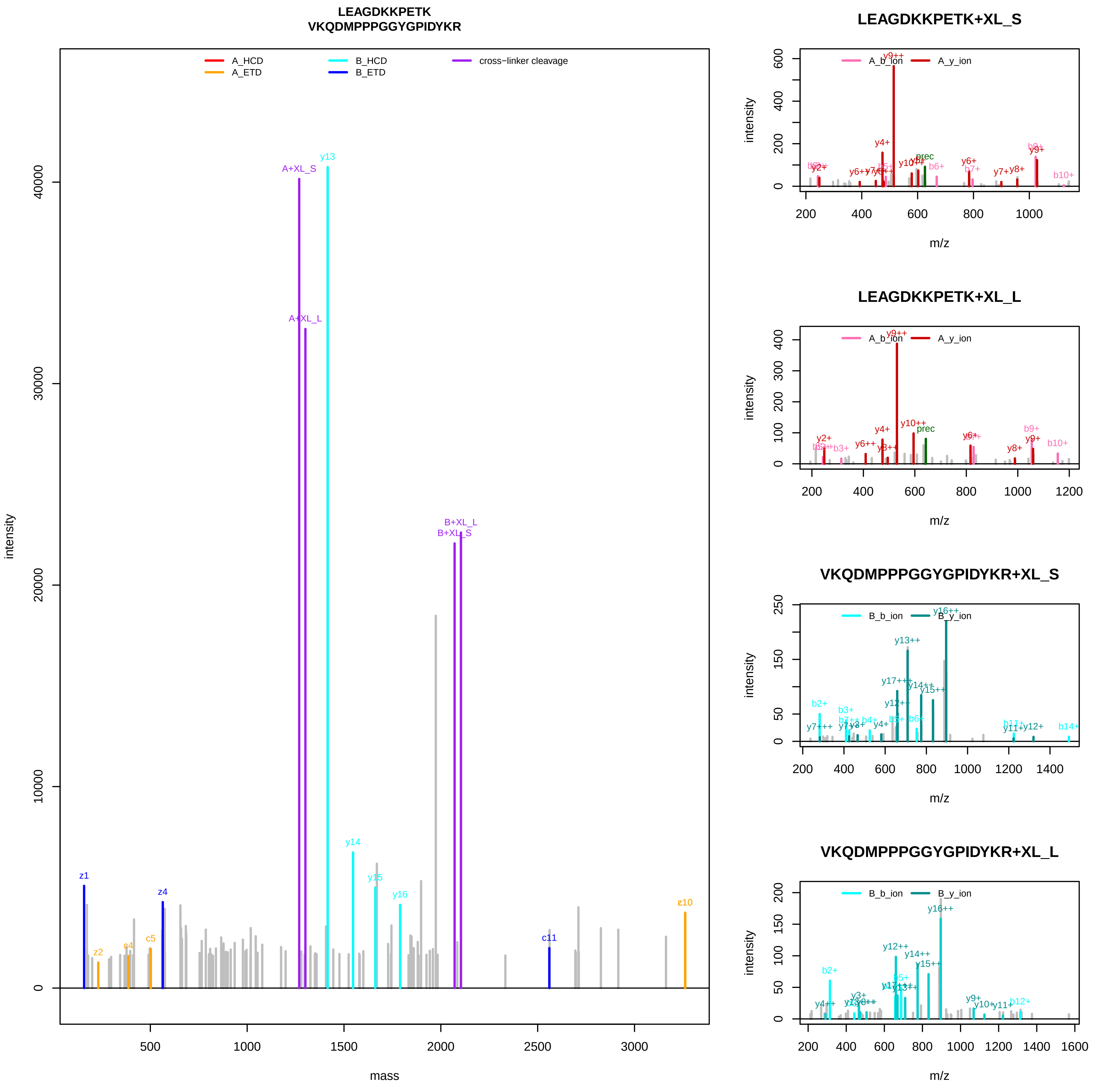

LEAGDKKPETK
VKQDMPPPGGYGPIDYKR
A_HCD
A_ETD
B_HCD
B_ETD
cross−linker cleavage
A+XL_S
A+XL_L
B+XL_L
B+XL_S
y13
y14
y15
y16
z1
z2
c4
c5
z4
c11
z10
intensity
mass
LEAGDKKPETK+XL_S
LEAGDKKPETK+XL_L
VKQDMPPPGGYGPIDYKR+XL_S
VKQDMPPPGGYGPIDYKR+XL_L
A_b_ion
A_y_ion
B_b_ion
B_y_ion
y9++
y4+
prec
y16++
y13++
y12++
y14++
y15++
b2+
m/z
